# Supplementary material for: Heat Shock Partially Dissociates the Overlapping Modules of the Yeast Protein-Protein Interaction Network: A Systems Level Model of Adaptation
Source: PLoS Comput Biol. 2011 Oct 13;7(10):e1002187. doi: 10.1371/journal.pcbi.1002187 (PMC3192799; doi:10.1371/journal.pcbi.1002187)
Supplement: Text S1 — This supporting information (Text S1) contains a detailed information on the distribution and variabilty of interaction weights, on correlation of mRNA abundances with unweighted degrees and on degree distributions of heat shocked yeast interactomes; a comparison of the metabolic networks of Buchnera aphidicola and Escherichia coli; additional data on the decrease of modular overlap in stresses other than heat shock and using other model parameters; as well as on the topological position of major bridges in the interactome in 8 supporting figures. The supporting information also contains the functional annotation of modules as well as the identity of major proteins with high community centrality and bridgeness values in 4 supporting tables. (PDF) [file pcbi.1002187.s001.pdf]

Supporting Information Text S1

Heat shock partially dissociates the overlapping modules of the yeast protein-protein interaction network: a systems level model of adaptation

Ágoston Mihalik and Peter Csermely*

Department of Medical Chemistry, Semmelweis University, Tűzoltó str. 37-47, H-1094 Budapest, Hungary

*E-mail: csermely@eok.sote.hu

Summary

This supporting information (Text S1) contains a detailed information on the distribution and variabilty of interaction weights, on correlation of mRNA abundances with unweighted degrees and on degree distributions of heat shocked yeast interactomes; a comparison of the metabolic networks of *Buchnera aphidicola* and *Escherichia coli*; additional data on the decrease of modular overlap in stresses other than heat shock and using other model parameters; as well as on the topological position of major bridges in the interactome in 8 supporting figures. The supporting information also contains the functional annotation of modules as well as the identity of major proteins with high community centrality and bridgeness values in 4 supporting tables.

Table of contents

Supporting Figures **3**
Figure S1. Cumulative distribution of protein-protein interaction weights of unstressed and heat shocked yeast cells. 3
Figure S2. Correlation of mRNA abundances with unweighted degrees of respective proteins in case of the top120 mRNA abundances of unstressed and heat shocked yeast cells. 4
Figure S3. Cumulative log-log distribution of weighted interactome degrees of unstressed and heat shocked yeast cells. 5
Figure S4. Metabolic networks of the symbiont, *Buchnera aphidicola* and the free living bacterium, *Escherichia coli*. 6
Figure S5. Decrease of modular overlap of the yeast interactome in different stress conditions. 7
Figure S6. Topological positions of proteins with persistent, or heat shock-induced bridgeness in the network of the strongest interactions of the yeast interactome. 8
Figure S7. Decrease of modular overlap of the yeast interactome using different model parameters. 9
Figure S8. Variability of interaction weights of unstressed yeast interactomes derived from relative changes of mRNA expression in different stress conditions. 10

Supporting Tables **11**
Table S1. Functional annotation of selected yeast interactome modules at different thresholds of core proteins. 11
Table S2. Functional annotation of yeast interactome modules in unstressed state and after heat shock. 12
Table S3. Yeast proteins with altered community centrality upon heat shock. 15
Table S4. Yeast proteins with altered bridgeness upon heat shock. 20

References **22**

Supporting Figures

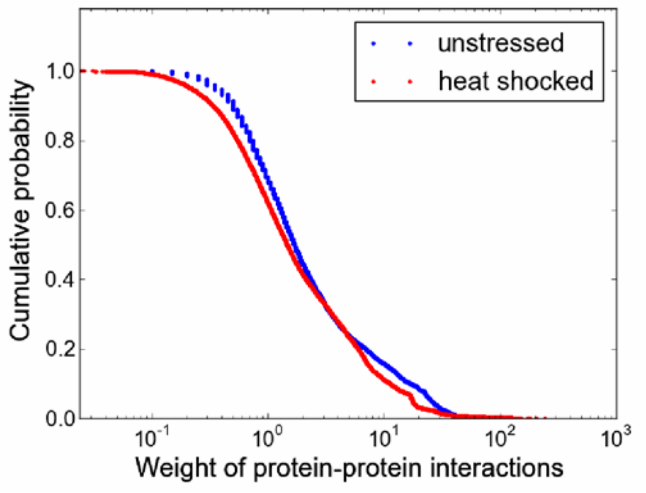

Figure S1. Cumulative distribution of protein-protein interaction weights of unstressed and heat shocked yeast cells. Interaction weights of yeast protein-protein interaction network (derived from the BioGRID database [1] as described in Methods of the main text) were generated by averaging of the mRNA abundances of the two interacting proteins. Unstressed and 15 min, 37°C heat shocked mRNA levels were obtained from the Holstege- [2] and Gasch-datasets [3], respectively as described in Methods of the main text. The cumulative distribution of unstressed and heat shocked interaction weights is shown using blue and red symbols, respectively. The distribution of interaction weights showed a significant shift towards lower weights upon heat shock (Wilcoxon paired test, $p<2.2*10^{-16}$).

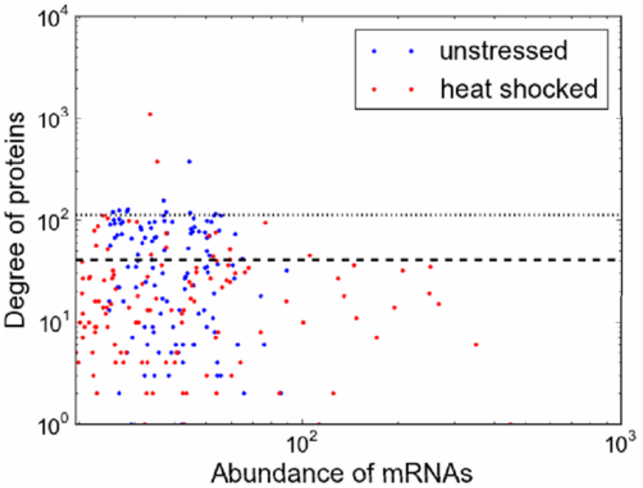

Figure S2. Number of unweighted hubs having the top 120 mRNA levels in unstressed and heat shocked yeast cells. Unstressed and heat shocked (15 min heat shock at 37°C) yeast mRNA levels were calculated and unweighted BioGRID protein-protein interaction networks were created as described in the legend of Figure S1 of Text S1 and in the Methods section of the main text. The top 120 mRNA abundances were selected and the unweighted degrees of the corresponding proteins were plotted as a function of their mRNA expression levels of unstressed (blue dots) and heat shocked (red dots) yeast cells. The dotted line shows the threshold of hubs set to the degree of 112 representing the top 1% of nodes having maximal unweigthed degrees in the whole interactome. The figure shows that 9 or 2 hubs were found among the proteins having the top 120 mRNA levels in unstressed or heat shocked yeast cells, respectively. The dashed line shows the top 10% of nodes, having 56 or 20 neighbor-rich nodes in unstressed or heat shocked yeast cells, respectively. Both data show that hubs associate with highly expressed mRNA (and protein) levels to a greater extent in unstressed than in heat shocked yeast cells.

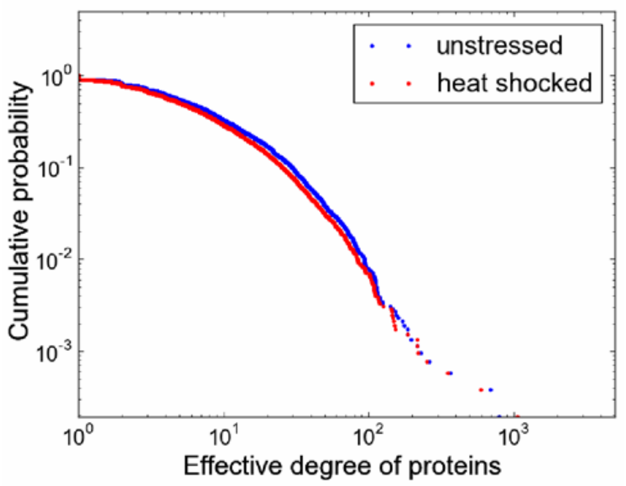

Figure S3. Cumulative log-log distribution of weighted interactome degrees of unstressed and heat shocked yeast cells. Unstressed and heat shocked (15 min heat shock at 37°C) yeast BioGRID protein-protein interaction networks were created as described in the legend of Figure S1 of Text S1 and in the Methods section of the main text. The effective degree was calculated as the effective number of weighted interactions of the respective node (see Methods of the main text for more details). The cumulative log-log distribution of unstressed and heat shocked effective degrees is shown using blue and red symbols, respectively. The distribution of effective degrees showed a scale-free like pattern and a significant shift towards lower degrees upon heat shock (Wilcoxon paired test, $p<2.2*10^{-16}$).

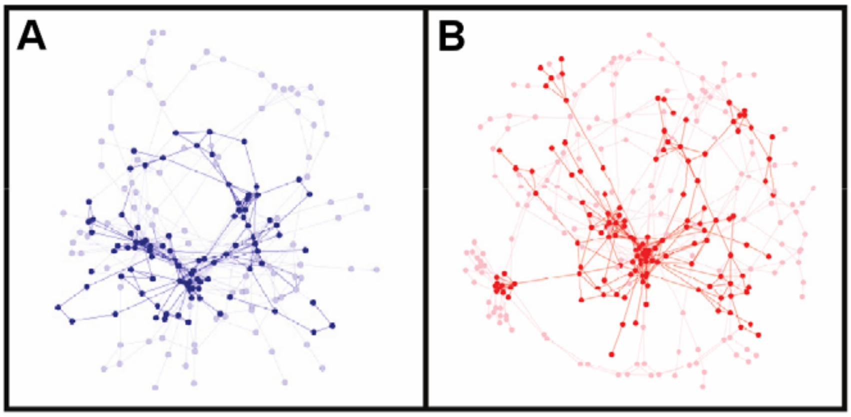

Figure S4. Metabolic networks of the symbiont, *Buchnera aphidicola* and the free living bacterium, *Escherichia coli*. Metabolic networks of *Buchnera aphidicola* (panel A) and *Escherichia coli* (panel B) were constructed based on the primary data of Thomas et al. [4] and Feist et al. [5], respectively. Frequent cofactors were deleted from the networks, except of those metabolic reactions, where cofactors were considered as main components. For the better comparison of networks, metabolic reactions were taken irreversible and flux balance analyses (FBA) were performed resulting in weighted networks. All flux quantities were minimized, whereas reactions non-affecting the biomass production were considered having zero flux. Weights were generated as the mean of the appropriate flux quantities in absolute value, except of the case when one of the fluxes was zero that resulted in a zero weight automatically. Subnetworks were created based on metabolic reactions having non-zero flux quantities, then giant components of the respective networks were visualized using the spring-embedded layout of Cytoscape [6]. Core reactions with weights being in the top 40% and nodes having at least one core interaction were labeled with darker colors. Panel A. Subnetwork of the metabolic network of *Buchnera aphidicola.* Panel B. Subnetwork of the metabolic network of *Escherichia coli*.

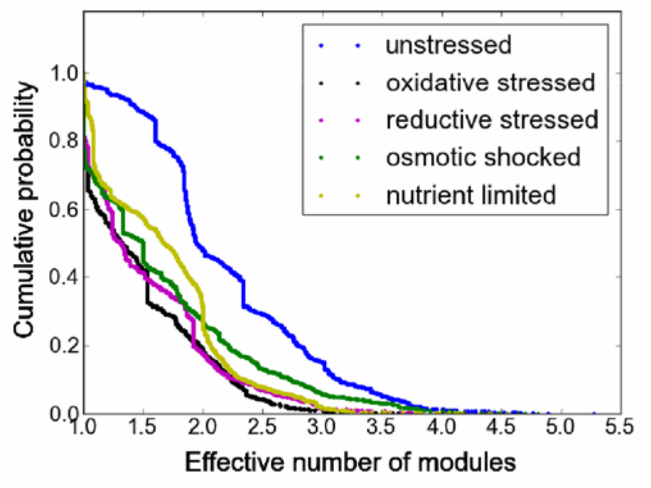

Figure S5. Decrease of modular overlap of the yeast interactome in different stress conditions. Protein-protein interaction weights of unstressed (blue dots), oxidative stressed (20 min menadione, black dots), reductive stressed (15 min dithiothreitol, magenta dots), osmotic shocked (15 min hypo-osmotic shock, green dots) and nutrient limited (0.5 h amino acid starvation, yellow dots) yeast BioGRID protein-protein interaction networks were created as described in the legend of Figure S1 of Text S1 and in the Methods section of the main text. Overlapping modules were calculated by the NodeLand influence function method combined with the ProportionalHill module membership assignment method [7] as described in Methods of the main text. The overlap of yeast interaction modules was represented by the cumulative distribution of the effective number of modules of yeast proteins (see Methods of the main text for more details). Upon different stress conditions (oxidative stress, reductive stress, osmotic shock and nutrient limitation) the effective number of modules, that a yeast protein simultaneously belongs to, was equally significantly decreased (Wilcoxon paired test, $p < 2.2*10^{-16}$ in all cases) similarly to the significant decrease of the same measure upon heat shock as showed in Figure 3 of the main text.

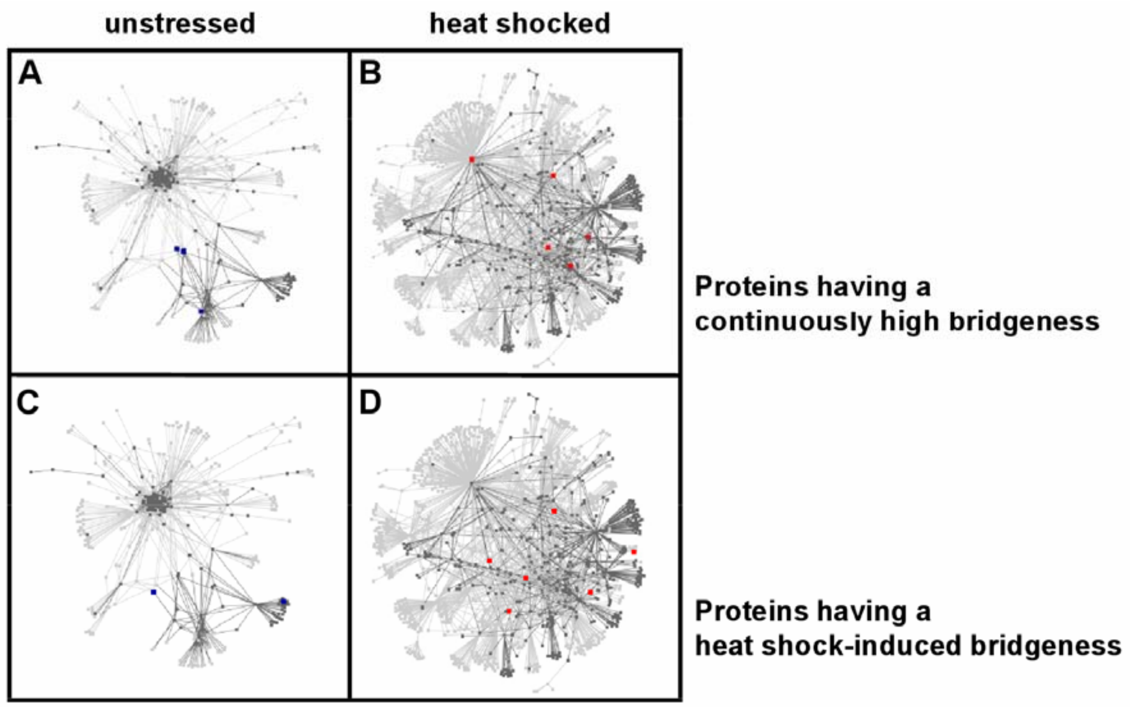

Figure S6. Topological positions of proteins with persistent, or heat shock-induced bridgeness in the network of the strongest interactions of the yeast interactome. Protein-protein interaction networks of unstressed (panels A and C) and heat shocked (15 min heat shock at 37°C; panels B and D) yeast cells were created as described in Methods of the main text. The subnetworks of their strongest links were determined and visualized as described in the legend of Figure 1 in the main text. Light grey colors denote the top 4%, while dark-grey colors the top 1% of interactions, respectively. Bridgeness of proteins was determined as described earlier [7]. Groups of proteins with persistent or heat shock-induced bridgeness (see Figure S6 and Table S4 of Text S1) are marked with larger blue filled circles in the unstressed conditions (panels A and C) and with larger red filled circles in the heat shocked conditions (panels B and D), respectively. Panels A and B. Topological positions of proteins having a persistently high bridgeness. Out of the 7 such proteins 3 and 5 proteins were visible in the subnetworks of the strongest links, before and after heat shock, respectively (see Figure 6 of the main text and Table S4 of Text S1). Panels C and D. Topological positions of proteins having a heat shock-induced bridgeness. Out of the 9 such proteins 2 and 6 proteins were visible in the subnetworks of the strongest links, before and after heat shock, respectively (see Figure 6 of the main text and Table S4 of Text S1). Bridges appeared at a larger ratio (31% compared to 69% before and after heat shock, respectively), and were re-organized to more inter-modular positions in the interactome of the strongest links after heat shock.

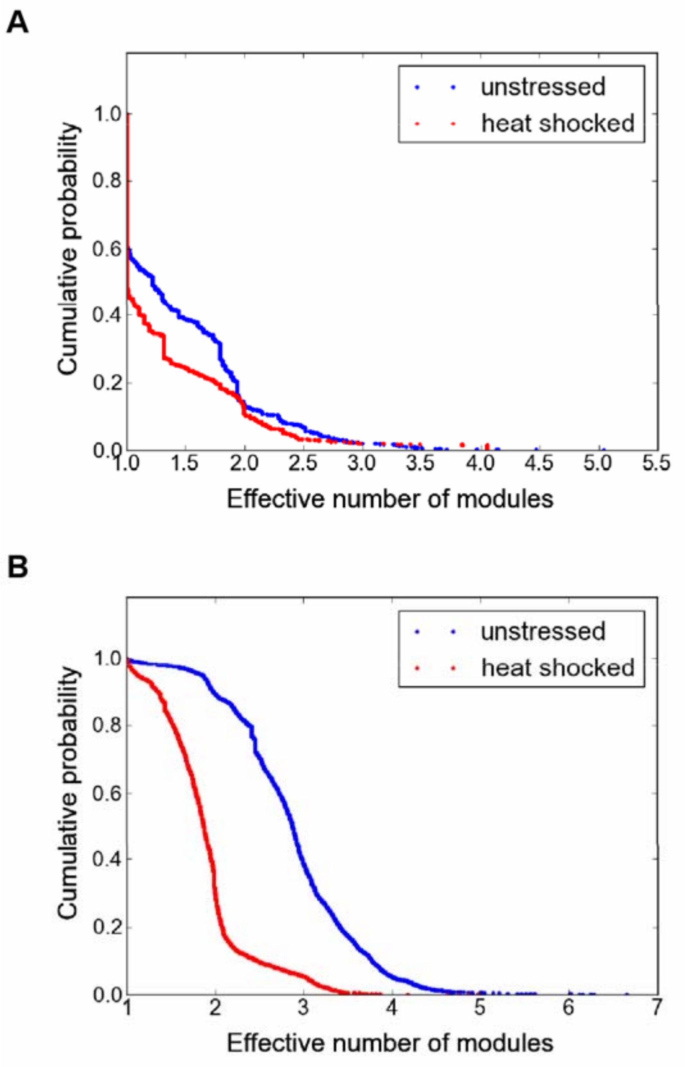

Figure S7. Decrease of modular overlap of the yeast interactome using different model parameters. Unstressed (blue dots) and heat shocked (15 min heat shock at 37°C, red dots) yeast protein-protein interaction networks using different model parameters were created as described in the Methods section of the main text. Overlapping modules were calculated by the NodeLand influence function method combined with the ProportionalHill module membership assignment method [7] as described in Methods of the main text. Panel A. Modular overlap of the high-confidence PPI dataset of Ekman et al. [8] in unstressed condition and upon heat shock. Here multiplication of the two node's mRNA abundances as link weights was used. The overlap of yeast interaction modules was represented by the cumulative distribution of the effective number of modules of yeast proteins (see Methods of the main text for more details). Upon heat shock the effective number of modules, that a yeast protein simultaneously belongs to, was significantly decreased (Wilcoxon paired test, $p < 2.2*10^{-16}$). Panel B. Modular overlap of the yeast BioGRID interactome in unstressed condition and upon heat shock, where mRNA expression data were logarithmically transformed. Here relative changes in mRNA levels were added to the baseline abundances and averaging of the two node's mRNA abundances as link weights was used (since logarithm transforms multiplication to addition). Upon heat shock the effective number of modules, that a yeast protein simultaneously belongs to, was significantly decreased (Wilcoxon paired test, $p < 2.2*10^{-16}$).

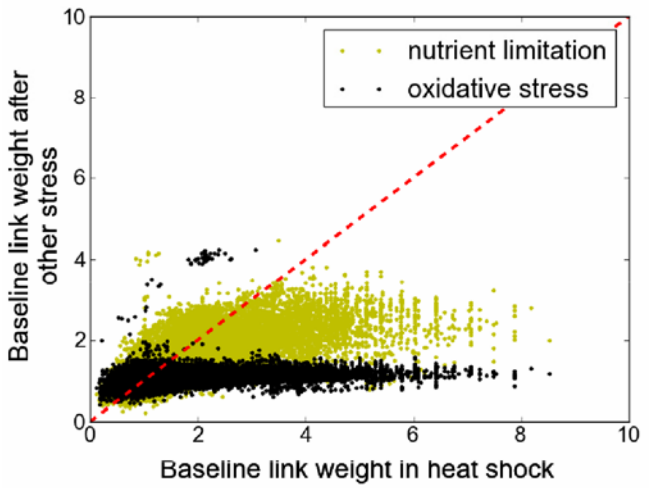

Figure S8. Variability of interaction weights of unstressed yeast interactomes derived from relative changes of mRNA expression in different stress conditions. Interaction weights of the yeast BioGRID protein-protein interactions networks (see the legend of Figure S1 of Text S1 and Methods of the main text for more details) were generated by using relative changes of mRNA expressions upon heat shock (15 min heat shock at 37°C), nutrient limitation (0.5 h amino acid starvation) and oxidative stress (20 min menadione). Weights were generated using a model, where relative changes upon stress were split to two changes: one-half for baseline weights and the other half for stressed weights (for more details see Methods section of the main text). Baseline interaction weights generated from changes upon heat shock were used as a reference, and the respective weights of nutrient limitation (yellow dots) and oxidative stress (black dots) were plotted as the function of the heat shock-related baseline interaction weights. The dashed red line represents a full correlation. The figure shows that oxidative stress- and nutrient limitation-related link weights are generally smaller than those in heat shock, which is due to the generally lower changes in mRNA levels in these two types of stresses than in heat shock.

Supporting Tables

Table S1. Functional annotation of selected yeast interactome modules at different thresholds of core proteins

GO ID	GO term: biological process	Number of core genes	Frequency of GO-term	p-value[a]	Threshold of core genes[b]
**A. An example of central interactome modules of unstressed yeast cells[c]**					
6412	translation	5 genes	5 out of 5 genes, 100.0%	$3.10*10^{-4}$	79.20%
		25 genes	25 out of 25 genes, 100.0%	$4.18*10^{-24}$	56.87%
		50 genes	50 out of 50 genes, 100.0%	$8.79*10^{-50}$	42.22%
		75 genes	73 out of 75 genes, 97.3%	$2.62*10^{-70}$	53.41%
		100 genes	81 out of 100 genes, 81.0%	$1.85*10^{-62}$	0.20%
**B. An example of central interactome modules of heat shocked yeast cells**					
6007	glucose catabolic process	5 genes	5 out of 5 genes, 100.0%	$1.31*10^{-10}$	49.01%
		25 genes	8 out of 25 genes, 32.0%	$6.80*10^{-11}$	4.56%
		50 genes	10 out of 50 genes, 20.0%	$2.01*10^{-11}$	0.27%
		75 genes	13 out of 75 genes, 17.3%	$1.81*10^{-14}$	0.12%
		100 genes	13 out of 100 genes, 13.0%	$9.92*10^{-13}$	0.07%

[a]Statistical analysis was performed by hypergeometric test.
[b]Threshold is defined as the lowest community centrality value divided by the highest community centrality value (in per cent) of the respective module core.
[c]The examples show the preservation of the original GO-term at a statistically significant manner as a wider and wider selection of modular constituents are examined.

Table S2. Functional annotation of yeast interactome modules in unstressed state and after heat shock

Module ranking[a]	Module centrality[b]	GO ID	GO term: biological process	p-value[c]	Top 5 gene names[d]
**A. Interactome modules of unstressed yeast cells**					
1	$7.16*10^{6}$	6412	translation	$3.10*10^{-4}$	YLR075W, YGL135W, YIL018W, YGR085C, YBL072C
2	$5.74*10^{6}$	6412	translation	$1.80*10^{-4}$	YBR191W, YGR085C, YGL103W, YNL178W, YBR031W
3	$1.72*10^{6}$	5975	carbohydrate metabolic process	$4.71*10^{-6}$	YKL060C, YCR012W, YHR174W, YGR192C, YOL086C
4	$2.13*10^{5}$	51276	chromosome organization	$4.25*10^{-5}$	YBR010W, YNL031C, YDR225W, YNL030W, YBL003C
5	$9.62*10^{4}$	51453	regulation of intracellular pH	$4.89*10^{-3}$	YBR106W, YHR026W, YGR060W, YLR372W, YEL027W
6	$1.08*10^{4}$	-	no significant biological process was found	$<1.00*10^{-2}$	YER117W, YBL187C, YER148W, YFR034C, YNL209W
7	$4.54*10^{3}$	7010	cytoskeleton organization[e]	$1.90*10^{-6}$	YFL039C, YLL050C, YHR179W, YBR109C, YLR291C[f]
8	$4.35*10^{3}$	43161	proteasomal ubiquitin-dependent protein catabolic process	$1.97*10^{-3}$	YDR328C, YHR021C, YLR167W, YDL126C, YMR276W
9	$4.09*10^{3}$	10499	proteasomal ubiquitin-independent protein catabolic process	$4.96*10^{-13}$	YER094C, YPR103W, YGR135W, YOL038W, YOR362C
10	$2.22*10^{3}$	48193	Golgi vesicle transport	$7.62*10^{-5}$	YDL192W, YDL137W, YPR110C, YKL196C, YDL226C
11	$1.70*10^{3}$	42254	ribosome biogenesis	$3.88*10^{-5}$	YKR057W, YGR285C, YER036C, YJL136C, YCL059C

Table S2. Functional annotation of yeast interactome modules in unstressed state and after heat shock (continued)

Module ranking[a]	Module centrality[b]	GO ID	GO term: biological process	p-value[c]	Top 5 gene names[d]
**A. Interactome modules of unstressed yeast cells** (continued)					
12	$8.88*10^{2}$	6360	transcription from RNA polymerase I promoter[g]	$5.82*10^{-10}$	YOR210W, YOR224C, YPR187W, YBR154C, YPR010C
13	$8.68*10^{2}$	7186	G protein coupled receptor protein signaling pathway[e]	$1.05*10^{-3}$	YGR037C, YFL026W, YLR359W, YBL079W, Q0110[h]
14	$8.20*10^{2}$	32543	mitochondrial translation	$3.81*10^{-8}$	YPL013C, YNL306W, YDR041W, YBR146W, YGR084C
15	$5.28*10^{2}$	51169	nuclear transport	$4.90*10^{-7}$	YDR002W, YLR293C, YMR235C, YGR218W, YKR048C
**B. Interactome modules of heat shocked yeast cells**					
1	$3.38*10^{6}$	10506	regulation of autophagy	$9.00*10^{-4}$	YDR343C, YDR342C, YLL039C, YKL203C, YJR066W
2	$2.88*10^{6}$	5975	carbohydrate metabolic process	$4.71*10^{-6}$	YCR012W, YKL060C, YGR192C, YHR174W, YOL086C
3	$4.15*10^{5}$	42026	protein refolding	$9.00*10^{-4}$	YLL026W, YOR027W, YLR216C, YOL013C, YOR244W
4	$2.37*10^{5}$	22402	cell cycle process	$5.00*10^{-3}$	YFL014W, YDR155C, YBR109C, YHR152W, YNL312W
5	$1.31*10^{5}$	6412	translation	$3.10*10^{-4}$	YDR382W, YLR075W, YGL103W, YDL083C, YOR063W
6	$5.55*10^{4}$	5992	trehalose biosynthetic process	$1.80*10^{-4}$	YBR126C, YMR251W, YCL040W, YML100W, YOL133W
7	$5.01*10^{4}$	-	no significant biological process was found	$<1.00*10^{-2}$	YNL160W, YBL032W, YOL133W, YDR134C, YNL055C
8	$3.55*10^{4}$	42026	protein refolding[e]	$5.36*10^{-5}$	YJR009C, YJL034W, YPR080W, YJL052W, YPL106C[i]

Table S2. Functional annotation of yeast interactome modules in unstressed state and after heat shock (continued)

Module ranking[a]	Module centrality[b]	GO ID	GO term: biological process	p-value[c]	Top 5 gene names[d]
**B. Interactome modules of heat shocked yeast cells** (continued)					
9	$3.45*10^4$	34284	response to monosaccharide stimulus	$8.20*10^{-4}$	YDR134C, YHR135C, YBL032W, YNL055C, YNL154C
10	$2.45*10^4$	48519	negative regulation of biological process	$6.48*10^{-5}$	YNL031C, YER177W, YBR010W, YDR099W, YOR244W
11	$3.49*10^3$	46467	membrane lipid biosynthetic process	$2.56*10^{-3}$	YDR276C, YDL212W, YBR036C, YBR183W, YDR307W
12	$2.36*10^3$	45454	cell redox homeostasis	$1.48*10^{-3}$	YLR109W, YGR209C, YIL035C, YJL141C, YMR059W
13	$1.10*10^3$	32543	mitochondrial translation	$1.07*10^{-5}$	YGR220C, YBL038W, YNL005C, YCR046C, YDR116C
14	$5.83*10^2$	55072	iron homeostasis	$7.93*10^{-5}$	YPL135W, YDR100W, YDL120W, YCL017C, YOL082W

[a]Module ranking is based on the community centrality value of the most central protein of the respective module.
[b]Module centrality is defined as community centrality value of the most central protein of the respective module.
[c]Statistical analysis was performed by hypergeometric test.
[d]Gene ORF names are listed in decreasing order of their community centrality values.
[e]GO term annotation from 8 genes.
[f]YLR429W, YGL106W, YNL138W are the additional 3 genes for annotation.
[g]Transcription from RNA polymerase III (GO ID:6383, $p=8.95*10^{-8}$) and II promoter (GO ID:6366, $p=1.18*10^{-3}$) are also significant for these genes.
[h]YKL130W, YHR005C, YOR212W are the additional 3 genes for annotation.
[i]YMR186W, YOR136W, YPL240C are the additional 3 genes for annotation.

Table S3. Yeast proteins with altered community centrality upon heat shock

ORF	Gene name	Functional annotation[a]
**A. small → large centrality[b] upon heat shock**		
**YBL036C**	YBL036C	Putative non-specific single-domain racemase based on structural similarity
**YCL040W**	GLK1	Glucokinase, catalyzes the phosphorylation of glucose at C6 in the first irreversible step of glucose metabolism
**YDR171W**	HSP42	Small heat shock protein (sHSP) with chaperone activity
**YDR342C**	HXT7	High-affinity glucose transporter of the major facilitator superfamily, nearly identical to Hxt6p, expressed at high basal levels relative to other HXTs, expression repressed by high glucose levels
**YDR343C**	HXT6	High-affinity glucose transporter of the major facilitator superfamily, nearly identical to Hxt7p, expressed at high basal levels relative to other HXTs, repression of expression by high glucose requires SNF3
**YER103W**	SSA4	Heat shock protein that is highly induced upon stress
**YER125W**	RSP5	E3 ubiquitin ligase of the NEDD4 family
**YFL014W**	HSP12	Plasma membrane localized protein that protects membranes from desiccation
**YJR066W**	TOR1	PIK-related protein kinase and rapamycin target
**YKL203C**	TOR2	PIK-related protein kinase and rapamycin target
**YLL026W**	HSP104	Heat shock protein that cooperates with Ydj1p (Hsp40) and Ssa1p (Hsp70) to refold and reactivate previously denatured, aggregated proteins
**YLR019W**	PSR2	Functionally redundant Psr1p homolog, a plasma membrane phosphatase involved in the general stress response
**YOL100W**	PKH2	Serine/threonine protein kinase involved in sphingolipid-mediated signaling pathway that controls endocytosis
**YOR181W**	LAS17	Actin assembly factor, activates the Arp2/3 protein complex that nucleates branched actin filaments
**B. large community centrality[b] in both conditions**		
**YCR012W**	PGK1	3-phosphoglycerate kinase, catalyzes transfer of high-energy phosphoryl groups from the acyl phosphate of 1,3-bisphosphoglycerate to ADP to produce ATP
**YDL229W**	SSB1	Cytoplasmic ATPase that is a ribosome-associated molecular chaperone, functions with J-protein partner Zuo1p
**YDR050C**	TPI1	Triose phosphate isomerase, abundant glycolytic enzyme
**YDR161W**	YDR161W	Putative protein of unknown function
**YDR188W**	CCT6	Subunit of the cytosolic chaperonin CCT ring complex, related to Tcp1p, essential protein that is required for the assembly of actin and tubulins in vivo
**YDR510W**	SMT3	Ubiquitin-like protein of the SUMO family, conjugated to lysine residues of target proteins
**YGR192C**	TDH3	Glyceraldehyde-3-phosphate dehydrogenase, isozyme 3, involved in glycolysis and gluconeogenesis
**YGR252W**	GCN5	Histone acetyltransferase, acetylates N-terminal lysines on histones H2B and H3
**YHR174W**	ENO2	Enolase II, a phosphopyruvate hydratase that catalyzes the conversion of 2-phosphoglycerate to phosphoenolpyruvate during glycolysis and the reverse reaction during gluconeogenesis
**YHR200W**	RPN10	Non-ATPase base subunit of the 19S regulatory particle (RP) of the 26S proteasome
**YKL060C**	FBA1	Fructose 1,6-bisphosphate aldolase, required for glycolysis and gluconeogenesis
**YKL152C**	GPM1	Tetrameric phosphoglycerate mutase, mediates the conversion of 3-phosphoglycerate to 2-phosphoglycerate during glycolysis and the reverse reaction during gluconeogenesis
**YLL039C**	UBI4	Ubiquitin, becomes conjugated to proteins, marking them for selective degradation via the ubiquitin-26S proteasome system

Table S3. Yeast proteins with altered community centrality upon heat shock (continued)

ORF	Gene name	Functional annotation[a]
**B. large community centrality[b] in both conditions** (continued)		
**YLR044C**	PDC1	Major of three pyruvate decarboxylase isozymes, key enzyme in alcoholic fermentation, decarboxylates pyruvate to acetaldehyde
**YNL127W**	FAR11	Protein involved in recovery from cell cycle arrest in response to pheromone, in a Far1p-independent pathway
**YOL086C**	ADH1	Alcohol dehydrogenase, fermentative isozyme active as homo- or hetero-tetramers
**YOR098C**	NUP1	Nuclear pore complex (NPC) subunit, involved in protein import/export and in export of RNAs, possible karyopherin release factor that accelerates release of karyopherin-cargo complexes after transport across NPC
**C. extra large → slightly smaller community centrality[b] upon heat shock**		
**YBL027W**	RPL19B	Protein component of the large (60S) ribosomal subunit, nearly identical to Rpl19Ap and has similarity to rat L19 ribosomal protein
**YBL072C**	RPS8A	Protein component of the small (40S) ribosomal subunit
**YBL092W**	RPL32	Protein component of the large (60S) ribosomal subunit, has similarity to rat L32 ribosomal protein
**YBR031W**	RPL4A	N-terminally acetylated protein component of the large (60S) ribosomal subunit, nearly identical to Rpl4Bp and has similarity to E. coli L4 and rat L4 ribosomal proteins
**YBR048W**	RPS11B	Protein component of the small (40S) ribosomal subunit
**YBR181C**	RPS6B	Protein component of the small (40S) ribosomal subunit
**YBR189W**	RPS9B	Protein component of the small (40S) ribosomal subunit
**YBR191W**	RPL21A	Protein component of the large (60S) ribosomal subunit, nearly identical to Rpl21Bp and has similarity to rat L21 ribosomal protein
**YCR031C**	RPS14A	Ribosomal protein 59 of the small subunit, required for ribosome assembly and 20S pre-rRNA processing
**YDL075W**	RPL31A	Protein component of the large (60S) ribosomal subunit, nearly identical to Rpl31Bp and has similarity to rat L31 ribosomal protein
**YDL082W**	RPL13A	Protein component of the large (60S) ribosomal subunit, nearly identical to Rpl13Bp
**YDL083C**	RPS16B	Protein component of the small (40S) ribosomal subunit
**YDL136W**	RPL35B	Protein component of the large (60S) ribosomal subunit, identical to Rpl35Ap and has similarity to rat L35 ribosomal protein
**YDR012W**	RPL4B	Protein component of the large (60S) ribosomal subunit, nearly identical to Rpl4Ap and has similarity to E. coli L4 and rat L4 ribosomal proteins
**YDR025W**	RPS11A	Protein component of the small (40S) ribosomal subunit
**YDR064W**	RPS13	Protein component of the small (40S) ribosomal subunit
**YDR382W**	RPP2B	Ribosomal protein P2 beta, a component of the ribosomal stalk, which is involved in the interaction between translational elongation factors and the ribosome
**YDR418W**	RPL12B	Protein component of the large (60S) ribosomal subunit, nearly identical to Rpl12Ap
**YDR447C**	RPS17B	Ribosomal protein 51 (rp51) of the small (40s) subunit
**YDR471W**	RPL27B	Protein component of the large (60S) ribosomal subunit, nearly identical to Rpl27Ap and has similarity to rat L27 ribosomal protein
**YER074W**	RPS24A	Protein component of the small (40S) ribosomal subunit
**YER102W**	RPS8B	Protein component of the small (40S) ribosomal subunit
**YFR031C-A**	RPL2A	Protein component of the large (60S) ribosomal subunit, identical to Rpl2Bp and has similarity to E. coli L2 and rat L8 ribosomal proteins
**YGL030W**	RPL30	Protein component of the large (60S) ribosomal subunit, has similarity to rat L30 ribosomal protein
**YGL031C**	RPL24A	Ribosomal protein L30 of the large (60S) ribosomal subunit, nearly identical to Rpl24Bp and has similarity to rat L24 ribosomal protein

Table S3. Yeast proteins with altered community centrality upon heat shock (continued)

ORF	Gene name	Functional annotation[a]
**C. extra large → slightly smaller community centrality[b] upon heat shock** (continued)		
**YGL076C**	RPL7A	Protein component of the large (60S) ribosomal subunit, nearly identical to Rpl7Bp and has similarity to E. coli L30 and rat L7 ribosomal proteins
**YGL103W**	RPL28	Ribosomal protein of the large (60S) ribosomal subunit, has similarity to E. coli L15 and rat L27a ribosomal proteins
**YGL135W**	RPL1B	N-terminally acetylated protein component of the large (60S) ribosomal subunit, nearly identical to Rpl1Ap and has similarity to E. coli L1 and rat L10a ribosomal proteins
**YGL147C**	RPL9A	Protein component of the large (60S) ribosomal subunit, nearly identical to Rpl9Bp and has similarity to E. coli L6 and rat L9 ribosomal proteins
**YGR034W**	RPL26B	Protein component of the large (60S) ribosomal subunit, nearly identical to Rpl26Ap and has similarity to E. coli L24 and rat L26 ribosomal proteins
**YGR085C**	RPL11B	Protein component of the large (60S) ribosomal subunit, nearly identical to Rpl11Ap
**YHL001W**	RPL14B	Protein component of the large (60S) ribosomal subunit, nearly identical to Rpl14Ap and has similarity to rat L14 ribosomal protein
**YHL033C**	RPL8A	Ribosomal protein L4 of the large (60S) ribosomal subunit, nearly identical to Rpl8Bp and has similarity to rat L7a ribosomal protein
**YHR141C**	RPL42B	Protein component of the large (60S) ribosomal subunit, identical to Rpl42Ap and has similarity to rat L44
**YHR203C**	RPS4B	Protein component of the small (40S) ribosomal subunit
**YIL018W**	RPL2B	Protein component of the large (60S) ribosomal subunit, identical to Rpl2Ap and has similarity to E. coli L2 and rat L8 ribosomal proteins
**YIL069C**	RPS24B	Protein component of the small (40S) ribosomal subunit
**YIL133C**	RPL16A	N-terminally acetylated protein component of the large (60S) ribosomal subunit, binds to 5.8 S rRNA
**YJL177W**	RPL17B	Protein component of the large (60S) ribosomal subunit, nearly identical to Rpl17Ap and has similarity to E. coli L22 and rat L17 ribosomal proteins
**YJL190C**	RPS22A	Protein component of the small (40S) ribosomal subunit
**YJR123W**	RPS5	Protein component of the small (40S) ribosomal subunit, the least basic of the non-acidic ribosomal proteins
**YJR145C**	RPS4A	Protein component of the small (40S) ribosomal subunit
**YKL180W**	RPL17A	Protein component of the large (60S) ribosomal subunit, nearly identical to Rpl17Bp and has similarity to E. coli L22 and rat L17 ribosomal proteins
**YLL045C**	RPL8B	Ribosomal protein L4 of the large (60S) ribosomal subunit, nearly identical to Rpl8Ap and has similarity to rat L7a ribosomal protein
**YLR029C**	RPL15A	Protein component of the large (60S) ribosomal subunit, nearly identical to Rpl15Bp and has similarity to rat L15 ribosomal protein
**YLR075W**	RPL10	Protein component of the large (60S) ribosomal subunit, responsible for joining the 40S and 60S subunits
**YLR340W**	RPP0	Conserved ribosomal protein P0 of the ribosomal stalk, which is involved in interaction between translational elongation factors and the ribosome
**YLR441C**	RPS1A	Ribosomal protein 10 (rp10) of the small (40S) subunit
**YLR448W**	RPL6B	Protein component of the large (60S) ribosomal subunit, has similarity to Rpl6Ap and to rat L6 ribosomal protein
**YML063W**	RPS1B	Ribosomal protein 10 (rp10) of the small (40S) subunit
**YML073C**	RPL6A	N-terminally acetylated protein component of the large (60S) ribosomal subunit, has similarity to Rpl6Bp and to rat L6 ribosomal protein
**YMR194W**	RPL36A	N-terminally acetylated protein component of the large (60S) ribosomal subunit, nearly identical to Rpl36Bp and has similarity to rat L36 ribosomal protein

Table S3. Yeast proteins with altered community centrality upon heat shock (continued)

ORF	Gene name	Functional annotation[a]
**C. extra large → slightly smaller community centrality[b] upon heat shock** (continued)		
**YMR242C**	RPL20A	Protein component of the large (60S) ribosomal subunit, nearly identical to Rpl20Bp and has similarity to rat L18a ribosomal protein
**YNL069C**	RPL16B	N-terminally acetylated protein component of the large (60S) ribosomal subunit, binds to 5.8 S rRNA
**YNL096C**	RPS7B	Protein component of the small (40S) ribosomal subunit, nearly identical to Rps7Ap
**YNL178W**	RPS3	Protein component of the small (40S) ribosomal subunit, has apurinic/apyrimidinic (AP) endonuclease activity
**YNL301C**	RPL18B	Protein component of the large (60S) ribosomal subunit, identical to Rpl18Ap and has similarity to rat L18 ribosomal protein
**YOL040C**	RPS15	Protein component of the small (40S) ribosomal subunit
**YOL127W**	RPL25	Primary rRNA-binding ribosomal protein component of the large (60S) ribosomal subunit, has similarity to E. coli L23 and rat L23a ribosomal proteins
**YOR063W**	RPL3	Protein component of the large (60S) ribosomal subunit, has similarity to E. coli L3 and rat L3 ribosomal proteins
**YOR096W**	RPS7A	Protein component of the small (40S) ribosomal subunit, nearly identical to Rps7Bp
**YOR312C**	RPL20B	Protein component of the large (60S) ribosomal subunit, nearly identical to Rpl20Ap and has similarity to rat L18a ribosomal protein
**YPL090C**	RPS6A	Protein component of the small (40S) ribosomal subunit
**YPL131W**	RPL5	Protein component of the large (60S) ribosomal subunit with similarity to E. coli L18 and rat L5 ribosomal proteins
**YPL198W**	RPL7B	Protein component of the large (60S) ribosomal subunit, nearly identical to Rpl7Ap and has similarity to E. coli L30 and rat L7 ribosomal proteins
**YPL220W**	RPL1A	N-terminally acetylated protein component of the large (60S) ribosomal subunit, nearly identical to Rpl1Bp and has similarity to E. coli L1 and rat L10a ribosomal proteins
**D. large → small community centrality[b] upon heat shock**		
**YDL014W**	NOP1	Nucleolar protein, component of the small subunit processome complex, which is required for processing of pre-18S rRNA
**YDR450W**	RPS18A	Protein component of the small (40S) ribosomal subunit
**YGR118W**	RPS23A	Ribosomal protein 28 (rp28) of the small (40S) ribosomal subunit, required for translational accuracy
**YHR010W**	RPL27A	Protein component of the large (60S) ribosomal subunit, nearly identical to Rpl27Bp and has similarity to rat L27 ribosomal protein
**YLR287C-A**	RPS30A	Protein component of the small (40S) ribosomal subunit
**YNL110C**	NOP15	Constituent of 66S pre-ribosomal particles, involved in 60S ribosomal subunit biogenesis
**YNL124W**	NAF1	RNA-binding protein required for the assembly of box H/ACA snoRNPs and thus for pre-rRNA processing, forms a complex with Shq1p and interacts with H/ACA snoRNP components Nhp2p and Cbf5p
**YNL132W**	KRE33	Essential protein, required for biogenesis of the small ribosomal subunit
**E. small community centrality[b] in both conditions**		
**YDR104C**	SPO71	Meiosis-specific protein of unknown function, required for spore wall formation during sporulation
**YDR222W**	YDR222W	Protein of unknown function
**YEL072W**	RMD6	Protein required for sporulation

Table S3. Yeast proteins with altered community centrality upon heat shock (continued)

ORF	Gene name	Functional annotation[a]
**E. small community centrality[b] in both conditions** (continued)		
**YFL056C**	AAD6	Putative aryl-alcohol dehydrogenase with similarity to *P. chrysosporium* aryl-alcohol dehydrogenase, involved in the oxidative stress response
**YGL263W**	COS12	Protein of unknown function, member of the DUP380 subfamily of conserved, often subtelomerically-encoded proteins
**YJR055W**	HIT1	Protein of unknown function, required for growth at high temperature
**YJR129C**	YJR129C	Putative protein of unknown function
**YLL052C**	AQY2	Water channel that mediates the transport of water across cell membranes, only expressed in proliferating cells, controlled by osmotic signals, may be involved in freeze tolerance
**YLR010C**	TEN1	Protein that regulates telomeric length
**YLR213C**	CRR1	Putative glycoside hydrolase of the spore wall envelope

[a]Functional annotation of yeast proteins was achieved by GO annotation database downloaded from http://www.geneontology.org/GO.downloads.annotations.shtml at 10/9/2010.

[b]Community centrality values of proteins were calculated by the NodeLand influence function method [7]. Groups are the same as on Figure 4 of the main text.

Table S4. Yeast proteins with altered bridgeness upon heat shock

ORF	Gene name	Functional annotation[a]
**The 4 most induced bridges of the 9 heat-induced bridges[b]**		
**YNL103W**	MET4	Leucine-zipper transcriptional activator, responsible for the regulation of the sulfur amino acid pathway, requires different combinations of the auxiliary factors Cbf1p, Met28p, Met31p and Met32p
**YNL189W****	SRP1	Karyopherin alpha homolog, forms a dimer with karyopherin beta Kap95p to mediate import of nuclear proteins, binds the nuclear localization signal of the substrate during import
**YNL197C***	WHI3	RNA binding protein that sequesters CLN3 mRNA in cytoplasmic foci
**YOL062C**	APM4	Mu2-like subunit of the clathrin associated protein complex (AP-2)
**The 5 less induced bridges of the 9 heat-induced bridges[b]**		
**YER021W***	RPN3	Essential, non-ATPase regulatory subunit of the 26S proteasome lid, similar to the p58 subunit of the human 26S proteasome
**YER125W***	RSP5	E3 ubiquitin ligase of the NEDD4 family
**YLR249W****	YEF3	Gamma subunit of translational elongation factor eEF1B, stimulates the binding of aminoacyl-tRNA (AA-tRNA) to ribosomes by releasing eEF1A (Tef1p/Tef2p) from the ribosomal complex
**YNL113W**	RPC19	RNA polymerase subunit AC19, common to RNA polymerases I and III
**YNL161W***	CBK1	Serine/threonine protein kinase that regulates cell morphogenesis pathways
**7 persistent bridges[b]**		
**YAR027W**	UIP3	Putative integral membrane protein of unknown function
**YBL032W***	HEK2	RNA binding protein involved in the asymmetric localization of ASH1 mRNA
**YDR510W****	SMT3	Ubiquitin-like protein of the SUMO family, conjugated to lysine residues of target proteins
**YJL092W***	SRS2	DNA helicase and DNA-dependent ATPase involved in DNA repair, needed for proper timing of commitment to meiotic recombination and transition from Meiosis I to II
**YLL039C****	UBI4	Ubiquitin, becomes conjugated to proteins, marking them for selective degradation via the ubiquitin-26S proteasome system
**YMR236W**	TAF9	Subunit (17 kDa) of TFIID and SAGA complexes, involved in RNA polymerase II transcription initiation and in chromatin modification, similar to histone H3
**YOL135C****	MED7	Subunit of the RNA polymerase II mediator complex
**18 persistent, albeit less dominant bridges[b]**		
**YAL024C**	LTE1	Protein similar to GDP/GTP exchange factors but without detectable GEF activity
**YBR009C**	HHF1	Histone H4, core histone protein required for chromatin assembly and chromosome function
**YBR017C**	KAP104	Transportin or cytosolic karyopherin beta 2
**YCL028W**	RNQ1	[PIN(+)] prion, an infectious protein conformation that is generally an ordered protein aggregate
**YDR188W**	CCT6	Subunit of the cytosolic chaperonin Cct ring complex, related to Tcp1p, essential protein that is required for the assembly of actin and tubulins in vivo
**YDR347W**	MRP1	Mitochondrial ribosomal protein of the small subunit
**YFR034C**	PHO4	Basic helix-loop-helix (bHLH) transcription factor of the myc-family
**YGR124W**	ASN2	Asparagine synthetase, isozyme of Asn1p
**YHR062C**	RPP1	Subunit of both RNase MRP, which cleaves pre-rRNA, and nuclear RNase P, which cleaves tRNA precursors to generate mature 5' ends
**YHR152W**	SPO12	Nucleolar protein of unknown function, positive regulator of mitotic exit
**YJR121W**	ATP2	Beta subunit of the F1 sector of mitochondrial F1F0 ATP synthase, which is a large, evolutionarily conserved enzyme complex required for ATP synthesis
**YKL203C**	TOR2	PIK-related protein kinase and rapamycin target

Table S4. Yeast proteins with altered bridgeness upon heat shock (continued)

ORF	Gene name	Functional annotation[a]
**18 persistent, albeit less dominant bridges[b]** (continued)		
**YLR150W**	STM1	Protein required for optimal translation under nutrient stress
**YLR340W**	RPP0	Conserved ribosomal protein P0 of the ribosomal stalk, which is involved in interaction between translational elongation factors and the ribosome
**YMR024W**	MRPL3	Mitochondrial ribosomal protein of the large subunit
**YOL027C**	MDM38	Mitochondrial inner membrane protein, involved in membrane integration of a subset of mitochondrial proteins
**YOR098C**	NUP1	Nuclear pore complex (NPC) subunit, involved in protein import/export and in export of RNAs, possible karyopherin release factor that accelerates release of karyopherin-cargo complexes after transport across NPC
**YPL178W**	CBC2	Small subunit of the heterodimeric cap binding complex that also contains Sto1p, component of the spliceosomal commitment complex
**The most decreased bridge of the 3 heat-decreased bridges[b]**		
**YNL110C**	NOP15	Constituent of 66S pre-ribosomal particles, involved in 60S ribosomal subunit biogenesis
**The 2 less decreased bridges of the 3 heat-decreased bridges[b]**		
**YDR172W**	SUP35	Translation termination factor eRF3
**YDR405W**	MRP20	Mitochondrial ribosomal protein of the large subunit

[a]Functional annotation of yeast proteins was achieved by GO annotation database downloaded from http://www.geneontology.org/GO.downloads.annotations.shtml at 10/9/2010.
[b]Bridgeness values of proteins were calculated as described earlier [7]. Groups are the same as on Figure 6 of the main text.
*Bridge appears as a node of the strongest links of the yeast interactome shown on Figure S6 in Text S1 in heat shock only.
** Bridge appears as a node of the strongest links of the yeast interactome shown on Figure S6 in Text S1 both in unstressed state and after heat shock.

References

1. Stark C, Breitkreutz BJ, Chatr-Aryamontri A, Boucher L, Oughtred R, et al. (2011) The BioGRID interaction database: 2011 update. Nucleic Acids Res 39: D698–704.
2. Holstege FC, Jennings EG, Wyrick JJ, Lee TI, Hengartner CJ, et al. (1998) Dissecting the regulatory circuitry of a eukaryotic genome. Cell 95: 717–728.
3. Gasch AP, Spellman PT, Kao CM, Carmel-Harel O, Eisen MB, et al. (2000) Genomic expression programs in the response of yeast cells to environmental changes. Mol Biol Cell 11: 4241–4257.
4. Thomas GH, Zucker J, Macdonald SJ, Sorokin A, Goryanin I, et al. (2009) A fragile metabolic network adapted for cooperation in the symbiotic bacterium Buchnera aphidicola. BMC Syst Biol 3: 24.
5. Feist AM, Henry CS, Reed JL, Krummenacker M, Joyce AR, et al. (2007) A genome-scale metabolic reconstruction for Escherichia coli K-12 MG1655 that accounts for 1260 ORFs and thermodynamic information. Mol Syst Biol 3: 121.
6. Shannon P, Markiel A, Ozier O, Baliga NS, Wang JT, et al. (2003) Cytoscape: a software environment for integrated models of biomolecular interaction networks. Genome Res 13: 2498–2504.
7. Kovacs IA, Palotai R, Szalay MS, Csermely P (2010) Community landscapes: an integrative approach to determine overlapping network module hierarchy, identify key nodes and predict network dynamics. PLoS ONE 5: e12528.
8. Ekman D, Light S, Bjorklund AK, Elofsson A (2006) What properties characterize the hub proteins of the protein-protein interaction network of *Saccharomyces cerevisiae*? Genome Biol 7: R45.
